# Genome sequence and annotation of *Myroides phaeus* DSM 23313^T^ isolated from a human oral cavity

**DOI:** 10.1128/mra.00565-26

**Published:** 2026-07-10

**Authors:** Farzad Ahmed, Nathalie Leon, Rebecca Roytman, Dimitrios Stamatis, Danielle Graham, Markus Göker, Vera Thiel, Rekha Seshadri, Wei-Jen Lin

**Affiliations:** 1 Biological Sciences Department, California State Polytechnic University 166593, Pomona, California, USA; 2 DOE Joint Genome Institute, Lawrence Berkeley National Laboratory 1666, Berkeley, California, USA; 3 Leibniz Institute DSMZ, German Collection of Microorganisms and Cell Cultures 28351, Braunschweig, Germany; Nanchang University, Nanchang, Jiangxi, China

**Keywords:** *Myroides phaeus*, DSM 23313, genome sequences

## Abstract

We present the genome sequence of *Myroides phaeus* DSM 23313^T^, a type strain isolated from a human oral cavity. The genome comprises 2.97 Mbp and contains 2,661 predicted genes. This genome analysis will provide insight into the multiple antibiotic resistance capabilities of this opportunistic pathogen.

## ANNOUNCEMENT

*Myroides phaeus* DSM 23313^T^, isolated from the oral cavity of a student at Weifang Medical University in China, is a gram-negative, aerobic bacterium of the family Flavobacteriaceae. It displays optimal growth at 28°C and pH 8.0 (1). Along with other *Myroides* species, *M. phaeus* can act as an opportunistic pathogen and is resistant to multiple antibiotics, requiring combination therapy to treat (2). The Joint Genome Institute sequenced this type strain as part of Phase II of the One Thousand Microbial Genomes (KMG-II) project, which aimed to increase the phylogenetic breadth of publicly available prokaryotic genomes (https://www.osti.gov/award-doi-service/biblio/10.46936/10.25585/60001024).

*M. phaeus* was retrieved from cryoculture (in 5% DMSO) and upscaled in three transfers to 1 L in Medium 545 (tryptic soy broth), followed by aerobic incubation at 100 rpm and 28°C for 24 h (https://mediadive.dsmz.de/medium/545). Genomic DNA was extracted using the MasterPure Gram-Positive DNA Purification Kit (Biosearch Technologies). Approximately 200 ng of DNA was sheared to ~300 bp using a Covaris LE220 focused ultrasonicator, size-selected by double-SPRI, and prepared for sequencing by end repair, A-tailing, and ligation with Illumina-compatible, uniquely barcoded IDT sequencing adaptors using the KAPA Library Preparation Kit (Roche KK8201). The quantified library was then multiplexed utilizing a TruSeq Paired-End Cluster Kit v4. Sequencing was performed on the Illumina HiSeq-2500 sequencer using HiSeq TruSeq SBS Sequencing Kits v4, following a 2 × 150 indexed run recipe. All raw reads were filtered using the default parameters of the BBtools suite v35.87 unless otherwise noted. BBDuk was run to trim adapters and remove reads ≤51 bp or with pre-trim average quality scores below eight or containing any “N” bases. BBMap was used to remove reads mapping to human, cat, dog, and mouse references, as well as common microbial contaminants, at ≥95% identity prior to sequence masking with BBMask (3, 4). No reads were filtered out by the processing, and a total of 4,885,032 reads were used for genome assembly with SPAdes v3.6.2 with parameters of --cov-cutoff auto --phred-offset 33 -t 8 -m 40 --careful -k 25,55,95 --12 (5). Contigs below 1 kbp were discarded, generating a final assembly of 60 contigs and 58 scaffolds with 265.4× coverage, 99.99% CheckM2 completeness, and 0.07% CheckM2 contamination. The genome was annotated by the IMG/M pipeline v4.11.2 (6, 7).

The genome of *Myroides phaeus* DSM 23313ᵀ contains 2,661 genes, 2,967,042 bp, and 33.19% GC (Table 1). Analysis with IMG/M KEGG pathways indicates the presence of multiple β-lactam resistance genes, including genes for RND efflux pumps, as well as a Class D β-lactamase that deactivates multiple β-lactams, including carbapenems (8–10). This β-lactamase gene (ID2677097265) exhibits >99% amino acid sequence identity to the curated OXA-347 β-lactamase (ARO3001777) in the CARD database (11). Interestingly, a proximal gene (ID2677097267) showed 97% sequence identity to a CARD-curated gene (ARO3004600) encoding lincosamide nucleotidyltransferase (LnuH), a novel class of lincosamide resistance first reported in *Riemerella* in 2018 (12). The genome sequences presented here provide a valuable resource for future investigations, especially into the genetic basis of its multidrug resistance.

**TABLE 1:** Genomic characteristics of *Myroides phaeus* DSM 23313^T^

Property	Data
Strain	DSM 23313^T^
Other strain designations	MY15, LMG 25566, JCM 17139
Country and year of isolation	China, 2009
Source	Oral cavity of a healthy student
Genome size (bp)	2,967,042
GC content (%)	33.19
Total genes	2,661
Protein-coding genes	2,564
rRNA-coding genes (5S, 16S, 23S)	4 (2, 1, 1)
tRNA-coding genes	87
CRISPR arrays	1
Total no. of reads	4,885,032
Read type	2 × 150 bp
No. of contigs	60
Contig N50 (bp)	115,267
Contig L50	9
No. of scaffolds	58
Scaffolds >50 kb	19
Scaffold N50 (bp)	120,613
Scaffold L50	9
Coverage	265.4×

## Data Availability

The genome sequence of *Myroides phaeus* DSM 23313^T^ has been deposited in the Joint Genome Institute’s Integrated Microbial Genomes & Microbiomes portal under IMG ID 2675903217. The genome sequence was also deposited at NCBI under these ID numbers: GenBank FNDQ00000000.1, Assembly GCA_900099675.1, Single Read Archive SRX2156222, Run SRR4233937, and BioProject PRJNA329885.
